# Mathematical Parameters of the COVID-19 Epidemic in Brazil and Evaluation of the Impact of Different Public Health Measures

**DOI:** 10.3390/biology9080220

**Published:** 2020-08-12

**Authors:** Renato M. Cotta, Carolina P. Naveira-Cotta, Pierre Magal

**Affiliations:** 1 General Directorate of Nuclear and Technological Development, DGDNTM, Brazilian Navy, Ilha das Cobras, Centro, Rio de Janeiro, RJ CEP 20091-000, Brazil; 2 Laboratory of Nano & Microfluidics and Microsystems, LabMEMS, Mechanical Engineering Department, POLI & COPPE, UFRJ, Federal University of Rio de Janeiro, Cidade Universitária, Rio de Janeiro, RJ CEP 21945-970, Brazil; carolina@mecanica.coppe.ufrj.br; 3 Institut de Mathématiques de Bordeaux, Université de Bordeaux, 351, COURS de la Libération, 33400 Talence, France; pierre.magal@u-bordeaux.fr

**Keywords:** epidemics modeling, SIRU model, Bayesian inference, MCMC, COVID-19, Brazil

## Abstract

A SIRU-type epidemic model is employed for the prediction of the COVID-19 epidemy evolution in Brazil, and analyze the influence of public health measures on simulating the control of this infectious disease. The proposed model allows for a time variable functional form of both the transmission rate and the fraction of asymptomatic infectious individuals that become reported symptomatic individuals, to reflect public health interventions, towards the epidemy control. An exponential analytical behavior for the accumulated reported cases evolution is assumed at the onset of the epidemy, for explicitly estimating initial conditions, while a Bayesian inference approach is adopted for the estimation of parameters by employing the direct problem model with the data from the first phase of the epidemy evolution, represented by the time series for the reported cases of infected individuals. The evolution of the COVID-19 epidemy in China is considered for validation purposes, by taking the first part of the dataset of accumulated reported infectious individuals to estimate the related parameters, and retaining the rest of the evolution data for direct comparison with the predicted results. Then, the available data on reported cases in Brazil from 15 February until 29 March, is used for estimating parameters and then predicting the first phase of the epidemy evolution from these initial conditions. The data for the reported cases in Brazil from 30 March until 23 April are reserved for validation of the model. Then, public health interventions are simulated, aimed at evaluating the effects on the disease spreading, by acting on both the transmission rate and the fraction of the total number of the symptomatic infectious individuals, considering time variable exponential behaviors for these two parameters. This first constructed model provides fairly accurate predictions up to day 65 below 5% relative deviation, when the data starts detaching from the theoretical curve. From the simulated public health intervention measures through five different scenarios, it was observed that a combination of careful control of the social distancing relaxation and improved sanitary habits, together with more intensive testing for isolation of symptomatic cases, is essential to achieve the overall control of the disease and avoid a second more strict social distancing intervention. Finally, the full dataset available by the completion of the present work is employed in redefining the model to yield updated epidemy evolution estimates.

## 1. Introduction

A new human coronavirus started spreading in Wuhan, China, by the end of 2019, and turned into a pandemic disease called COVID-19 as declared by the World Health Organization on 11 March 2020. Since then, the affected countries and cities around the world have been reacting in different ways, towards locally controlling the disease evolution. These measures include general isolation through quarantine and massive testing for focused isolation, with varying degrees of success so far, as can be analyzed from the limited data available. Naturally, China offers the earliest and longest time series on reported infectious cases and the resulting effects of combining different public health interventions. As of 26 March 2020, there were no reports in China of further internal contaminations, and all the new cases were associated with infected individuals that (re)entered in the country. Despite the apparent success of the interventions in China, each region or country might require a specific combination of measures, due to demographic spatial distribution and age structure, health system capabilities, and social-economical characteristics. In this sense, it urges to have a mathematical model that would allow for the simulation of such possible interventions on the epidemy evolution within the short and long terms. This article presents a collaborative research effort towards the construction of an epidemy evolution prediction tool, which combines direct and inverse problem analysis and is both reliable and easy to implement and execute. This work was initially motivated towards offering some insight into the control of COVID-19 within Brazil since its early stage of evolution.

The classical susceptible-infectious-recovered (SIR) model describes the transmission of diseases between susceptible and infectious individuals and provides the basic framework for almost all phenomenological epidemic models. At the onset of the coronavirus epidemy in China, there were some initial studies for the prediction of its evolution and the analysis of the impact of public health measures [1], which however did not consider in the modeling the presence of unreported infectious individuals cases, which are in practice inherent to this process. The present work is thus based on the SIRU-type model proposed in Reference [2], which deals with the epidemic outbreak in Wuhan by introducing the unreported cases in the modeling, and evaluating the consequences of public health interventions. This was a direct application of previous developments [3,4] on the fundamental problem of parameter estimation in mathematical epidemic models, accounting for unreported symptomatic infectious individuals. This same modeling approach was later on employed in the analysis of the epidemic outbreak in different countries, including China, South Korea, Germany, Italy, United Kingdom, Spain, and France [5,6,7,8]. Besides identifying unreported cases, this simple and robust model also introduces a latency period and a time variable transmission rate, which can simulate a public health orientation change, such as in a general isolation measure. In addition, an exponential analytical behavior is assumed for the accumulated reported cases evolution along with an initial phase just following the onset of the epidemy, which, upon fitting of the available data, allows for the explicit analytical estimation of the transmission rate and the associated initial conditions required by the model.

Here, the SIRU-type model in References [2,3,4,5,6,7,8,9,10,11] is implemented for the direct problem formulation of the COVID-19 epidemy evolution, adding a time variable parametrization for the fraction of asymptomatic infectious that become reported symptomatic individuals, a very important parameter in the public health measure associated with massive testing and consequent focused isolation. The same analytical identification procedure is maintained for the required initial conditions, as obtained from the early stage exponential behavior assumption [2,3,4,5,6,7,8]. However, a Bayesian inference approach is here adopted for parametric estimations, complementing these analytical estimates obtained through the hypothesis of exponential growth in the early stage of the epidemy, before any public health intervention. Other recent contributions have successfully combined phenomenological deterministic compartmental models with Bayesian inference approaches for state estimation and parameter estimation [12,13,14,15,16]. For the present purposes, the Markov Chain Monte Carlo method with the Metropolis-Hastings sampling algorithm has been employed [17,18,19,20,21]. At first, the goal of the inverse problem analysis was estimating the parameters associated with the transmission rate and the fraction of asymptomatic infectious that become reported symptomatic individuals, which can be quite different in the various regions and countries and may also vary according to the public health measures. Then, in light of the success in this parametrization and on the estimation of the required coefficients, an extended estimation was also implemented which incorporates the average time the asymptomatic infectious are asymptomatic and the average time the infectious stay in the symptomatic condition, due to the noticeable uncertainty on these parameters in the medical literature. The proposed approach was then applied to the data from China, initially by taking just the first portion of these data points in the estimation, while using the second portion to validate the model using the estimated parameters with just the first phase of the epidemy evolution, and later on by employing the whole time series in the MCMC estimation procedure, thus identifying parameters for the whole evolution period. This second estimation was particularly aimed at refining the data for the average times that asymptomatic infectious individuals and symptomatic individuals remain infectious. Upon validation of the approach through the data for China, we have proceeded to the analysis of the epidemy dynamics in Brazil, employing 35 days (15 February until 29 March) of collected information on reported symptomatic infectious individuals. First, this initial phase data was employed in the estimation of parameters, followed by the prediction of the epidemy evolution in Brazil. For this purpose, the following days (up to day 60) were reserved to be used in the validation of the proposed model for the COVID-19 evolution in Brazil during this initial phase. Then, we have explored the time variation of both the transmission rate and the fraction of asymptomatic infections that become reported symptomatic individuals, to reflect public health interventions, in simulating possible government measures, as described in different scenarios. Finally, the full dataset of reported cases available by completion of this work, was employed in the redefinition of the model to provide predictions of the epidemy evolution based on more recent observations at that particular moment.

## 2. The Forward SIRU-Type Model

The implemented SIRU-type model [2,3,4,5,6,7,8] is given by the following initial value problem:

(1a) $$\frac{dS\left(t\right)}{dt}=-\tau \left(t\right)S\left(t\right)\left[I\left(t\right)+U\left(t\right)\right]$$

(1b) $$\frac{dI\left(t\right)}{dt}=\tau \left(t\right)S\left(t\right)\left[I\left(t\right)+U\left(t\right)\right]-\nu I\left(t\right)$$

(1c) $$\frac{dR\left(t\right)}{dt}=\nu_{1}\left(t\right)I\left(t\right)-\eta R\left(t\right)$$

(1d) $$\frac{dU\left(t\right)}{dt}=\nu_{2}\left(t\right)I\left(t\right)-\eta U\left(t\right)$$

where,

(2a) $$\nu_{1}\left(t\right)=\nu f\left(t\right)$$

(2b) $$\nu_{2}\left(t\right)=\nu \left(1-f\left(t\right)\right)$$

with initial conditions

(3a) $$S\left(t_{0}\right)=S_{0}$$

(3b) $$I\left(t_{0}\right)=I_{0}$$

(3c) $$R\left(t_{0}\right)=0$$

(3d) $$U\left(t_{0}\right)=U_{0}$$

**Figure 1:**
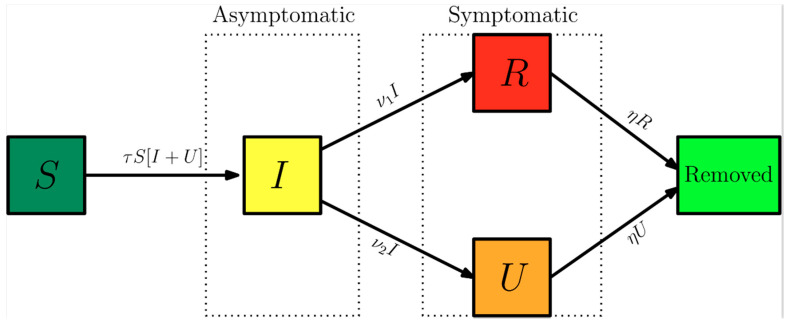
Flow chart illustrating the infection path process [3].

Here, *t_0_* is the beginning date of the epidemy in days, *S*(*t*) is the number of individuals susceptible to infection at time t, *I*(*t*) is the number of asymptomatic infectious individuals at time t, *R*(*t*) is the number of reported symptomatic infectious individuals (i.e., symptomatic infections with mild to severe symptoms) at time t, and *U*(*t*) is the number of unreported symptomatic infectious individuals (i.e., symptomatic infections with light to mild symptoms) at time t. Asymptomatic infectious individuals *I*(*t*) are infectious for an average period of 1/*ν* days. Reported symptomatic individuals *R*(*t*) are infectious for an average period of 1/*η* days, as are unreported symptomatic individuals *U*(*t*). We assume that reported symptomatic infectious individuals *R*(*t*) are reported and isolated immediately and cause no further infections. The asymptomatic individuals *I*(*t*) can also be viewed as having a low-level symptomatic state. All infections are acquired from either *I*(*t*) or *U*(*t*) individuals. The fraction *f*(*t*) of asymptomatic infections become reported symptomatic infections, and the fraction 1 − *f*(*t*) becomes unreported symptomatic infections. The rate that asymptomatic infectious become reported symptomatic is ν_1_(*t*) = *f*(*t*)ν, while the rate that asymptomatic infections become unreported symptomatic is *ν*_2_(*t*) = (1 − *f*(*t*)) *ν*, where *ν*_1_(*t*) + *ν*_2_(*t*) = *ν*. The transmission rate, *τ*(*t*), is also allowed to be a time variable function along the evolution process. Figure 1 below illustrates the infection process as a flow chart.

The time variable coefficients, *τ*(*t*) and *f*(*t*), are chosen to be expressed as:

(4a) $$\tau \left(t\right)=\tau_{0}\;,\;0\leq t\leq N$$

(4b) $$\tau \left(t\right)=\tau_{0}\exp\left(-\mu \left(t-N\right)\right),\;t>N$$

(4c) $$f\left(t\right)=f_{0}\;,\;0\leq t\leq N_{f}$$

(4d) $$f\left(t\right)=\left(f_{max-}f_{0}\right)\left[1-\exp\left(-\mu_{f}\left(t-N_{f}\right)\right)\right]+f_{0},\;t>N_{f}$$

These parametrized functions are particularly useful in interpreting the effects of public health interventions. For instance, the transmission rate, τ(t), is particularly affected by a reduced circulation achieved through a general isolation or quarantine measure or by the relaxation of this social distancing, while the fraction f(t) of asymptomatic infections that become reported, thus isolated, cases can be drastically increased by a massive testing measure with focused isolation. In the above relations, μ is the attenuation factor for the transmission rate, N is the starting time in days for application of the public health intervention to change transmission rate, while $\mu_{f}$ is the argument of the *f*(*t*) variation between the limits ($f_{0},\;f_{max}$). The first time variable function has been previously considered in References [5,6,7,8], while the second one has been introduced in the present work, to allow for the examination of combined measures. Existence and uniqueness of positive solutions for nonlinear SIRU-type models, which include system (1) as a special case, have been more closely discussed in References [22,23]. Moreover, it should be recalled that the present class of ordinary differential deterministic model is more appropriate to regions with fairly homogeneous spatial behavior and characteristics, since the spatial spread of epidemic disease in a geographical setting [23] is not explicitly accounted for in the present work.

The cumulative number of reported cases at time *t*, CR(t), which is the quantity offered by the actual available data, and the a priori unknown cumulative number of unreported cases, CU(t), are given by:

(5a) $$CR\left(t\right)=\int_{t_{0}}^{t}\nu_{1}\left(s\right)I\left(s\right)ds$$

(5b) $$CU\left(t\right)=\int_{t_{0}}^{t}\nu_{2}\left(s\right)I\left(s\right)ds$$

The daily number of reported cases from the model, DR(t), can be obtained by computing the solution of the following equation:

(6a) $$\frac{dDR\left(t\right)}{dt}=\nu f\left(t\right)I\left(t\right)-DR\left(t\right)$$

with initial conditions

(6b) $$DR\left(t_{0}\right)=DR_{0}$$

Both Equation (1a–d) and Equation (6a,b) are here numerically solved through the mixed symbolic-numerical *Mathematica* v.12 platform [24], using the built-in function NDSolve, with prescribed relative and absolute errors control to within 10 digits. This routine provides interpolating function objects as output, thus offering a continuous representation of the dependent variables at the completion of the computations. These interpolating functions are then analytically integrated through the Integrate function [24] to yield accurate results for the accumulated reported and unreported cases, Equation (5a,b).

## 3. The Backward SIRU-Type Model

Inverse problem analysis is nowadays a common practice in various science and engineering contexts, in which the groups involved with experiments and numerical simulation collaborate to obtain the maximum information from the available data, towards the best possible use of the modeling for the problem under study. Here, as mentioned in the introduction, we first review an analytical parametric estimation described in more details in References [4,5,6,7,8], that from the initial phase of the epidemy evolution allows to explicitly obtain the unknown initial conditions of the model, while offering a reliable estimate for the transmission rate at the onset of the epidemy. Nevertheless, even after these estimates, a few other parameters in the model remain unknown, either due to the specific characteristics of the physical conditions or response to the epidemy in each specific region, or due to lack of epidemiological information on the disease itself. Therefore, an inverse problem analysis was undertaken aimed at estimating the main parameters involved in the model, as summarized in Table 1 below. In case 1, which is associated with the dataset on the accumulated reported cases for China, the focus is on the parametrized time variation of the transmission rate ($\tau_{0}\;\mathrm{and}\;\mu$), while the fraction of asymptomatic infectious individuals that become reported ($f_{0}$), in this case, it was assumed constant. Then, in case 2, the inverse problem analysis was extended to refine the information on the average times (1/*ν* and 1/*η*) through a simultaneous estimation of the five parameters. Next, in case 3, employing the dataset for Brazil just up to 29 March, the parametrized time variation of the transmission rate ($\tau_{0}\;\mathrm{and}\;\mu$) and the fraction of asymptomatic infectious individuals that become reported f(t), assumed time-variable, are estimated by parametrization of an abrupt variation that requires just the estimation of $f_{max}\;\mathrm{and}\;N_{f}$. Finally, in case 4, the model was redefined for the second stage of the epidemy evolution in Brazil, through estimation of five parameters associated with new time-variable functions for the transmission rate and partition coefficient along this second phase.

The statistical inversion approach here implemented falls within the Bayesian statistical framework [8,9,10,11,12], in which (probability distribution) models for the measurements and the unknowns are constructed separately and explicitly, as shall be briefly reviewed in what follows.

As explained in previous works employing this model [4,5,6,7,8], it is assumed that in the early phase of the epidemy, the cumulative number of reported cases grows approximately exponentially, according to the following functional form:

(7a) $$CR\left(t\right)=\chi_{1\;}\exp\left(\chi_{2\;}t\right)-\chi_{3\;},\;t\geq t_{0}$$

After fitting this function to the early stage of the epidemy evolution, one may extract the information on the unknown initial conditions, in the form [4,5,6,7]:

(7b) $$t_{0}=\frac{1}{\chi_{2}}[\ln(\chi_{3\;})-\ln\left(\chi_{1}\right)]$$

(7c) $$I_{0}=\frac{\chi_{3}\chi_{2}}{f_{0}\nu }$$

(7d) $$U_{0}=\frac{\left(1-f_{0}\right)\nu }{\eta +\chi_{2}}I_{0}$$

In addition, an excellent estimate for the initial transmission rate can be obtained from the same fitted function, in the form:

(7e) $$\tau_{0}=\frac{\chi_{2}+\nu }{S_{0}}\frac{\eta +\chi_{2}}{\left(1-f_{0}\right)\nu +\eta +\chi_{2}}$$

Moreover, the basic reproductive number for this initial phase model is estimated as:

(7f) $${ℛ}_{0}=\frac{\tau_{0}S_{0}}{\nu }\left[1+\frac{\left(1-f_{0}\right)\nu }{\eta }\right]$$

The statistical approach for the solution of inverse problems here adopted employs the Metropolis-Hastings algorithm for the implementation of the Markov chain Monte Carlo (MCMC) method [8,9]. The MCMC method is used in conjunction with the numerical solution of the ordinary differential system, Equations (1)–(3), for estimating the remaining model parameters. Consider the vector of parameters appearing in the physical model formulation as:

**P***^T^* ≡ [*P*_1_, *P*_2_, …, *P_NP_*] (8)

is available (**Y**) containing the measurements *Y_m_* at time *t_m_*, *m* = 1, …, *M.*

Bayes’ theorem can then be stated as [8,9]:

(9) $$\pi_{posterior}\left(P\right)=\pi \left(P|Y\right)=\frac{\pi_{prior}\left(P\right)\pi \left(Y|P\right)}{\pi \left(Y\right)}$$

where $\pi_{posterior}\left(P\right)$ is the posterior probability density, that is, the conditional probability of the parameters **P** given the measurements **Y**,$\;\pi_{prior}\left(P\right)$ is the prior density, that is, the coded information about the parameters prior to the measurements, π(Y|P) is the likelihood function, which expresses the likelihood of different measurement outcomes **Y** with **P** given, and π(Y) is the marginal probability density of the measurements, which plays the role of a normalizing constant. If different *prior* probability densities are assumed for the parameters, the posterior probability distribution may not allow an analytical treatment. In this case, Markov chain Monte Carlo (MCMC) methods are used to draw samples of all possible parameters, and thus inference on the posterior probability becomes inference on the samples [8,9]. Throughout this work, the mathematical formulation is supposed to perfectly represent the physical problem, and the measurement errors are assumed to be Gaussian random variables, with zero means, known covariance matrix W and independent of the parameters P. The measurement errors, ε**,** are assumed to be additive, that is:

(10) Y=CR+ε

where, CR is the vector of solutions of the mathematical formulation (calculated through Equation (5a)), obtained with the vector of parameters P. Due to the additive model for the measurement errors the likelihood function, that gives the relative probability density of different measurement outcomes Y with a fixed P can be written as proportional to:

(11) $$\pi \left(Y|P\right)\propto e^{-\frac{1}{2}{\left(Y-CR\right)}^{T}W^{-1}\left(Y-CR\right)}.$$

A posterior probability, in Bayesian statistics, can be understood as the “revised” probability of the prior information, after taking into consideration the measurements. However, in this work, it is unfeasible to obtain an analytical posterior distribution through the Bayes theorem, due to the non-linearity of the model and the large space of parameters involved. Thus, the MCMC method is able to provide a picture of the posterior distribution, without solving the mathematical integrals in Bayes’ rule. The idea is to approximate the posterior distribution by a large collection of samples of values. 

The idea behind the Metropolis-Hasting sampling algorithm is illustrated below, and these steps should be repeated until it is judged that a sufficiently representative sample has been generated:

(1) Start the chain with an initial value, that usually comes from any prior information that one may have;
(2) Randomly generate a proposed jump aiming that the chain will move around and efficiently explores the region of the parameter space. The proposal distribution can take on many different forms, in this work a Gaussian random walk was employed, implying that the proposed jumps will usually be near the current one;
(3) Compute the probability of moving from the current value to the proposed one. Candidates moving to regions of higher probability will be definitely accepted. Candidates in regions of lower probability can be accepted only probabilistically. If the proposed jump is rejected, the current value is tally again. For more details on theoretical aspects of the Metropolis-Hastings algorithm and MCMC methods and its application, the reader should refer to References [8,9,10,11,12].

## 4. Results

### 4.1. Model Validation: China

**Figure 2:**
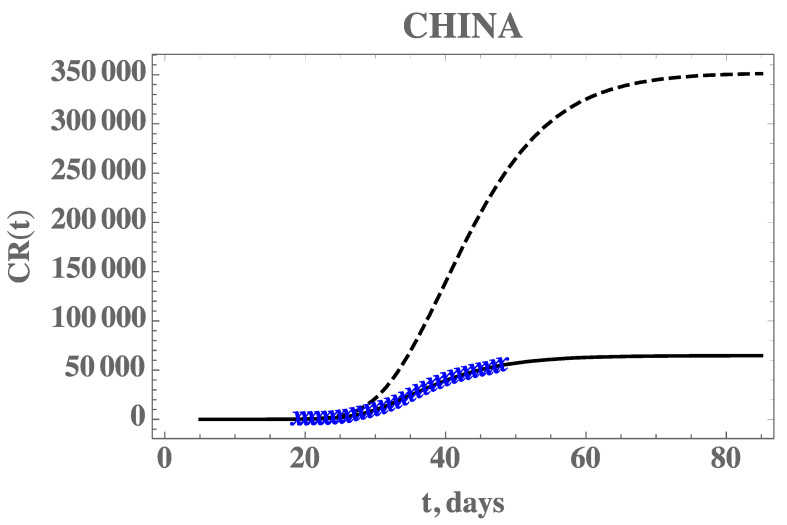
Comparison of the theoretical model for reported cases, CR(t), with the initial guesses from Table 2 (black dashed line), against the model prediction with the estimated values from Table 3 (solid black line), and actual data from China from 19 January to 17 February (blue cross)—Case 1: CH3p.

Before proceeding to the analysis of the COVID-19 epidemy evolution within Brazil, the proposed direct-inverse problem analysis approach is validated. In this sense, due to the availability of the earliest dataset on this pandemic, we have chosen to use the information from China in terms of the accumulated reported infectious cases. The data for China was extracted from Reference [6], complemented by the more recent data from Reference [25] from 1 January up to 17 April 2020. The exponential fit for the early phase of the China CR(t) dataset provided the estimates of the three parameters, $\chi_{1}=0.14936,\;\chi_{2}=0.37680,\;\chi_{3}=1.0,$ from which we have estimated $t_{0}=5.046$. The remaining data for the initial conditions, $I_{0}\;\mathrm{and}\;U_{0}$, and the early stage transmission rate, $\tau_{0}$, are in fact recalculated from within the MCMC algorithm, since the changing values of *f* will affect them, according to Equation (7c–e). The average times in the model were first taken as 1/*ν* = 7 and 1/*η* = 7 days and the isolation measures were taken at *n* = 25 days [6]. First, experimental data from China from the period of 19 January up to 17 February was employed in demonstrating the estimation of three parameters, $f_{0},\;\mu ,\;\mathrm{and}\;\tau_{0},$ assuming there is no significant time variation in the function *f*(*t*) ($\mu_{f}=0$). In the absence of more informative priors, uniform distributions were employed for all three parameters under estimation. Table 2 presents the prior information and the initial guesses for the parameters. If the initial guesses were used to predict the CR(t) behavior, a marked over-estimation of the accumulated reported infected individuals would occur, especially in the long term, as can be noticed in Figure 2, confirming the need for a proper parameter estimation, as will be shown.

The central tendency (mean value) of the posteriors here sampled, after neglecting the first 20,000 burning in states of the chain, are called the estimated values. Both the estimated values and their 99% confidence intervals are presented in Table 3. It should be mentioned that these values are fairly close to those employed in Reference [6], where τ_0_ was estimated as 4.51 × 10^−8^. Once a value of *f_0_* = 0.8 was assumed, which means that 20% of symptomatic infectious cases go unreported, it led to a good agreement with the data by taking *μ* = 0.139 in Reference [6].

**Figure 3:**
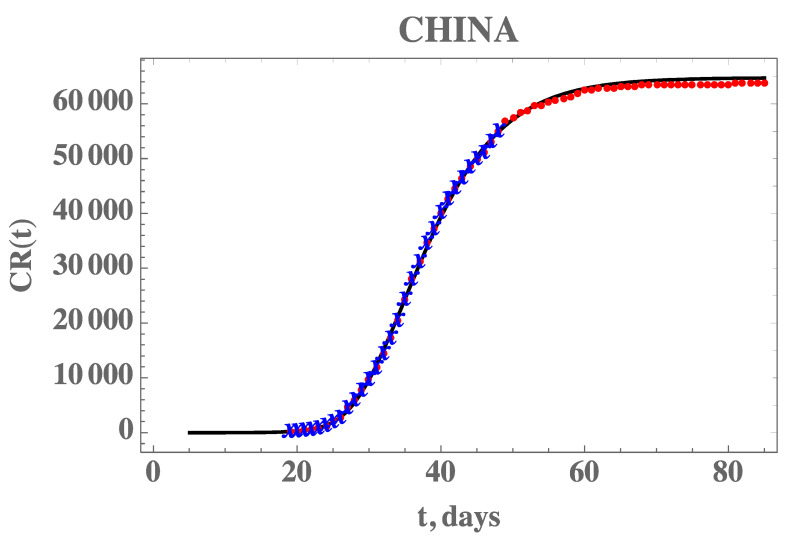
Comparison of the theoretical model for CR(t) with the three estimated parameter values from Table 3 (solid line), against the dataset for China from 19 January to 17 February (blue stars) and from 18 February to 16 April (red dots)—Case 1: CH3p.

Figure 2 also demonstrates the adherence of the model with the data within this portion of the dataset, once the estimated values in Table 3 are employed in the forward problem solution, as can be seen from the excellent agreement between the estimated CR(t) (solid line) and the experimental data from China (blue stars). The desired model validation is illustrated in Figure 3, confirming the excellent agreement of China’s full dataset (period from 19 January until 16 April) with the mathematical model predictions, after adopting the estimated values for the parameters in Table 3. It should be recalled that non-informative priors were adopted for the three parameters, as presented in Table 2, and except for the transmission rate, when Equation (7e) provides an excellent initial guess, the remaining guesses were completely arbitrary. A uniform prior means that all possible values, in the specified limits, are equally likely to be sampled, i.e., no prior information can be distinguished among possible values.

Although the presently estimated parameters have led to a good prediction of the second phase of the China epidemy evolution data, there are still uncertainties associated with the average times here assumed both equal to seven days, according to Reference [6]. This choice was based on early observations of the infected asymptomatic and symptomatic patients in Wuhan, but more recent studies have been refining the information on the epidemy evolution and the disease itself, such as in References [26,27,28]. For this reason, we have also implemented a statistical inverse analysis with the full dataset of China, but now seeking the estimation of five parameters, to simultaneously estimate the average times (1/*ν* and 1/*η*). Both uniform and Gaussian distributions were adopted for the two new parameters, with initial guesses of 1/*ν* = 7 days and 1/*η* = 7 days, and *n* = 25 days, as employed in Reference [6]. Table 4 presents the prior information and the initial guesses for the parameters.

**Figure 4:**
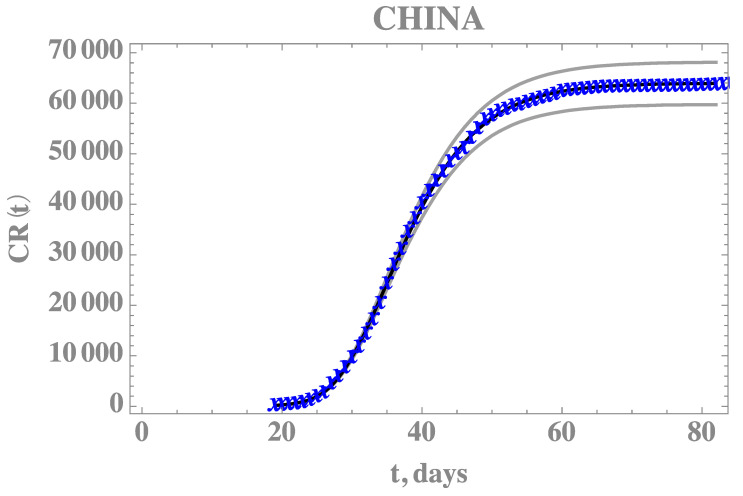
Comparison of the theoretical model for CR(t) with the five estimated parameter values (solid black line) and 99% confidence intervals (gray lines), against the complete dataset for China from 19 January up to 16 April (blue cross)—Case 2: CH5p.

Table 5 provides the estimated values and 99% confidence intervals for all five parameters, with Gaussian priors for the two average times with data obtained from References [26,27,28], after neglecting the first 15,000 burning in states of the chain. The most affected parameter in comparison with the previous estimates is the average time 1/η, which is also the one with the widest confidence interval. This behavior is also evident from the Markov chains for this parameter, now simultaneously estimated. Figure 4 compares the theoretical predictions with the model incorporating the five estimated parameters as in Table 5, against the full CR(t) dataset for China, confirming the improved agreement. The 99% confidence interval bounds for this predicted behavior is also shown in Figure 4, bounded by the grey lines.

### 4.2. Model Application: Brazil

The CR(t) data for the accumulated reported infectious in Brazil, from 25 February (day 1), when the first infected individual was reported, up to 29 June (day 127), is presented in Appendix A. Similarly to the previous example with the data from China, a portion of the available data on accumulated reported infectious was employed in the estimation of model parameters, up to 29 March (day 35), when the present study was initiated. Then, a second portion of the data, from 30 March (day 36) up to 23 April (day 60), was utilized in verifying this first constructed model. 

Following the hybrid procedure described above, the exponential phase of the evolution was first fitted, taking the data from day 10 to 25, yielding the estimates of the three parameters, $\chi_{1}=0.42552,\;\chi_{2}=0.293696,\;\chi_{3}=3.2335,$ from which we have estimated $t_{0}=6.9051$. The remaining data for the initial conditions, $I_{0}\;\mathrm{and}\;U_{0}$, and the early stage transmission rate, $\tau_{0}$, are in fact recalculated from within the MCMC algorithm, since the changing values of $f_{0}$ will affect them, according to Equation (7c–e). The average times in the model were taken as 1/*ν* = 6.20798 days and 1/*η* = 11.2784 days, which were obtained from the MCMC simulation on the full dataset for China (Table 5), as discussed in the previous section.

The Brazilian states governments took isolation measures starting on *n* = 21 days, which was enforced by most states and municipalities throughout the country within a few days. Moreover, there were initially only 30,000 exam kits available, and an additional 30,000 were later acquired. However, until mid-April at least, the resulting rather small ration of testing per million inhabitants in Brazil and the retardation in the exam results confirmation, due to a centralized operation, has caused a perceptible change in the data structure for the reported infectious cases, which can only be represented by a time-varying function *f*(*t*). The progressive reduction on the number of executed exams of the symptomatic individuals and the delay of the results availability, has certainly affected the partition of reported to unreported cases by the end of this period covered by the dataset for this first stage. Therefore, the more general model, including the time variation of the partition *f*(*t*), and Equation (4c,d), was here implemented for a more refined inverse problem analysis. It is then expected that a reduction on the f value can be identified ($f_{max}<f_{0}$), with an abrupt variation on the exponential behavior, here assumed as a sharp, functional time dependence (large $\mu_{f}$). Therefore, a statistical inverse problem analysis is undertaken, this time for estimating five parameters, namely, $f_{0},\;\mu ,\;\tau_{0},\;f_{max},\;\mathrm{and}\;N_{f}\;$(Case 3—BR5p) aimed at enhancing the overall agreement with the CR(t) data behavior, with a likely reduction on the partition of the reported and unreported infectious cases.

With uniform distributions for all five parameters, guided by the previous estimates for the first three parameters, and arbitrary guesses for $f_{max},\;\mathrm{and}\;N_{f}$, the prior distributions and initial guesses for the five parameters are presented in Table 6 and the five estimated quantities, after neglecting the first 80,000 burning in states of the chain are shown in Table 7, together with the 99% confidence interval for each parameter.

**Figure 5:**
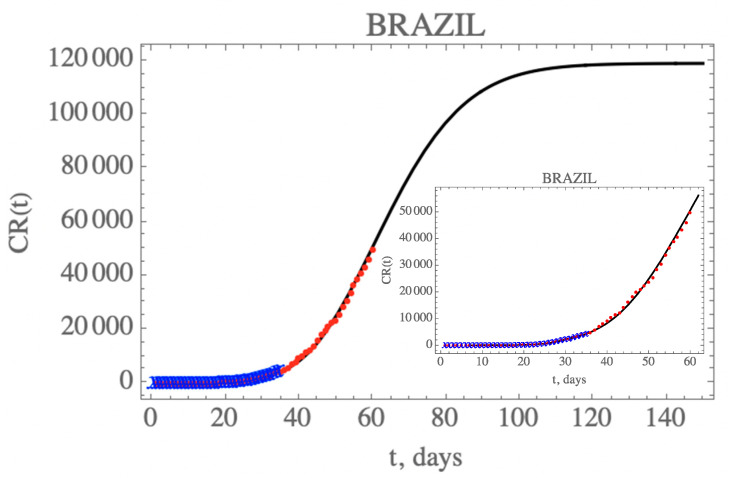
Prediction of the accumulated reported infectious, CR(t), with the five estimated parameter values from the available dataset for Brazil from 25 February up to 29 March (blue cross) and validated with the data up to 23 April (red dots).

Figure 5 presents the predicted evolution of the accumulated reported infectious cases in Brazil, CR(t), from 25 February up to day 150, plotted as the solid black line. Also shown in this figure are the blue crosses in the first portion of the available data, up to 29 March, which were employed in the estimation of the parameters in Table 7, that compose this initial model. In addition, the red dots represent the second portion of the available data from 30 March until 23 April (day 60), that were not employed in the parametric estimation, but were saved for model validation. It is clear that the built model has an excellent predictive feature, reproducing the epidemy evolution up to the end of the reserved data for validation (up to day 60), with a relative error below 5.8% during this first phase of the epidemy evolution.

**Figure 6:**
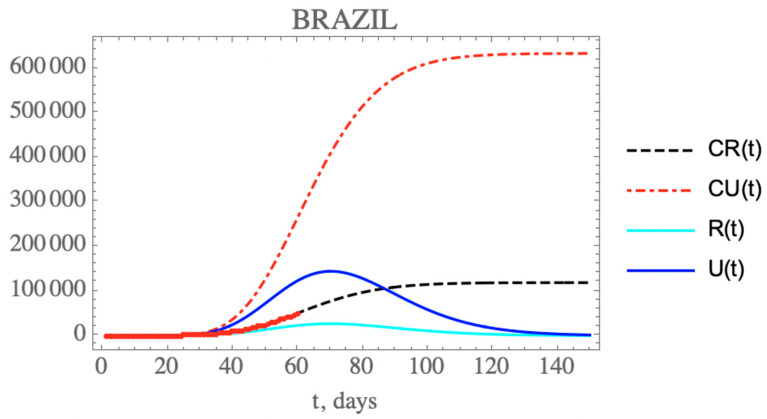
Comparison of the theoretical model for CR(t) (dashed black curve), CU(t) (dashed red curve), R(t) (solid cyan curve) and U(t) (solid blue curve) with the five estimated parameter values from the available dataset for Brazil from 25 February up to 29 March. (red dots show available data of CR(t) up to 23 April, day 60).

One can see the marked reduction on the *f*(*t*) parameter from the estimates in Table 7, which results in the increase of the unreported to reported infectious cases, as is shown in Figure 6 for CR(t) and CU(t) predictions up to 150 days. Clearly, the reduction on the testing, and thus, on the isolation of reported infectious individuals, leads to an increase on the total number of infected symptomatic individuals after 150 days (752,888 cases), including unreported (633,698) and reported cases (119,190). Both the reported and unreported infectious individual curves, R(t) and U(t), show a peak at around the 70th day (3 May). Naturally, these projections are inherently assuming that no new interventions or changes in public health would occur from day 35 on, including circulation alterations and more intensive testing, or otherwise, the estimated parameters would not represent such a new phenomenological situation.

The initial phase estimation was repeated, starting with the first 35 data points, and successively implementing another extra 10 points, up to 55 data points. The final behavior of the reported cases to the graphical scale remains essentially the same, reproducing quite well the remaining available data up to around day 65. Therefore, the essential phenomenological characteristics of this initial phase are captured by the adopted 35 points estimates, and was basically confirmed by increasing the number of considered data points. As will be shown in what follows, though this initially constructed model led to an accurate prediction of the actual epidemy evolution even up to around day 65, the data for accumulated reported cases started detaching from the theoretical curve after this period, yielding much higher values after a few weeks. For this reason, it is crucial to implement a scenarios analysis to anticipate such a behavior, based on likely measures or unplanned changes in public health, as will be discussed in the next section.

### 4.3. Scenarios Analyses: Brazil

Next, the model constructed with this initial parametric estimation is employed in the prediction of the COVID-19 evolution in Brazil under different hypothesis. By the end of this initial period of 60 days, though the proposed model with parameters estimated from the data up to day 35 gave very good predictions, it was already evident that the circulation in the country, as observed from the COVID-19 mobility reports made available [29,30], was progressively increasing in comparison to the first few weeks after the isolation measures, with respect to workplaces and groceries/pharmacies, especially associated with private vehicles and pedestrian circulation. This tendency becomes more evident after the Easter holiday, corresponding to day 50 onward, and it could be expected that a reduction on the transmissivity attenuation factor would result. Besides, the first set of 500,000 testing kits from a planned amount of 10 million [31], finally arrived in Brazil by day 60, with an expected impact on increasing the partition factor, and thus, on increasing the number of reported cases with respect to unreported ones, in the following days. Thus, the prospective scenarios here proposed are aimed at interpreting the epidemy evolution as a result of these two alterations in public health.

Five scenarios were here explored: (i) This is the base case above proposed, assuming that the initial public health interventions remain effective, with the same transmission rate decay due to isolation and the same low partition parameter identified through the first 35 data points; (ii) due to the progressive reduction on part of the circulation modes, a reduction on the transmission rate decay parameter is implemented after day 50, but maintaining the same testing capability as before; (iii) the same reduction on the transmission rate decay parameter of the previous scenario is kept, but now with an increase in the partition coefficient representing an enhanced testing capability starting at day 64; (iv) the transmission rate decay parameter is further reduced, to explore the influence of further circulation increase, while keeping the same fraction of reported cases of the previous scenario, through a more intensive testing; (v) a combination of interventions leading to further increasing the transmission rate by reducing the decay parameter, but simultaneously further increasing the conversion factor of unreported to reported cases with respect to the initially estimated value, *f*_0_.

Table 8 summarizes both the fixed and variable parameter values adopted for these five scenarios. The additional public health interventions simulated in the above scenarios act on either the time variation of the transmission coefficient or on the reported to unreported partition coefficient. The parametric changes are assumed to start at chosen dates further ahead of the initial set of data (day 35), in the present case t = 50 and 64 days (*N*_2_ = 50; *N*_*f*2_ = 64), respectively, for acting on the coefficients, τ(t) and f(t), according to the following parametrizations:

(12a) $$\tau \left(t\right)=\tau_{0}\;,\;0\leq t\leq N$$

(12b) $$\tau \left(t\right)=\tau_{0}\exp\left(-\mu \left(t-N\right)\right),\;N<t\leq N_{2}$$

(12c) $$\tau \left(t\right)=\tau_{02}\exp\left(-\mu_{2}\left(t-N_{2}\right)\right),\;t>N_{2}$$

with

(12d) $$\tau_{02}=\tau_{0}\exp\left(-\mu \left(N_{2}-N\right)\right)$$

(12e) $$f\left(t\right)=f_{0}\;,\;0\leq t\leq N_{f}$$

(12f) $$f\left(t\right)=\left(f_{max-}f_{0}\right)\left[1-\exp\left(-\mu_{f}\left(t-N_{f}\right)\right)\right]+f_{0},\;N_{f}<t\leq N_{f2}$$

(12g) $$f\left(t\right)=\left(f_{max2-}f_{02}\right)\left[1-\exp\left(-\mu_{f2}\left(t-N_{f2}\right)\right)\right]+f_{02},\;t>N_{f2}$$

with

(12h) $$f_{02}=\left(f_{max-}f_{0}\right)\left[1-\exp\left(-\mu_{f}\left(N_{f2}-N_{f}\right)\right)\right]+f_{0}$$

In the first scenario, it is assumed that no additional public health interventions are implemented, other than those already reflected by the data up to 29 March (day 35), which would then be fully maintained throughout the control period, and the epidemy should evolve from that date, under the parameters above identified. Figure 5 has already shown the evolution of the accumulated and instantaneous reported, CR(t) and R(t), unreported, CU(t) and U(t), infectious individuals, up to 150 days. Due to the fairly low value of *f*(*t*) starting with $f_{0}\approx 0.30$ and dropping to $f_{max}\approx 0.16,$ in light of the fairly low number of PCR kits made available and other implementation difficulties during this initial phase, the accumulated number of reported infectious cases is quite low with respect to the unreported ones, as already discussed. No predictions on casualties are here proposed, since these are highly dependent on age structure, social-economic conditions, and health system response. It should be recalled that this initial period of 35 days reflects the strict initial quarantine that was applied at least at the most affected regions, starting in day 21, which is confirmed through the global mobility reports [29,30]. However, these are ideal conditions that would be rather unlikely to be enforced for a much longer period, due to the sociological and economic characteristics of the country. As will be discussed more closely in what follows, the available mobility reports later on made available have in fact clearly indicated that especially after the Easter holiday weekend (day 50 and on), the circulation was progressively increased in average terms within the country, despite the quarantine measures retained by most of the federation states.

**Figure 7:**
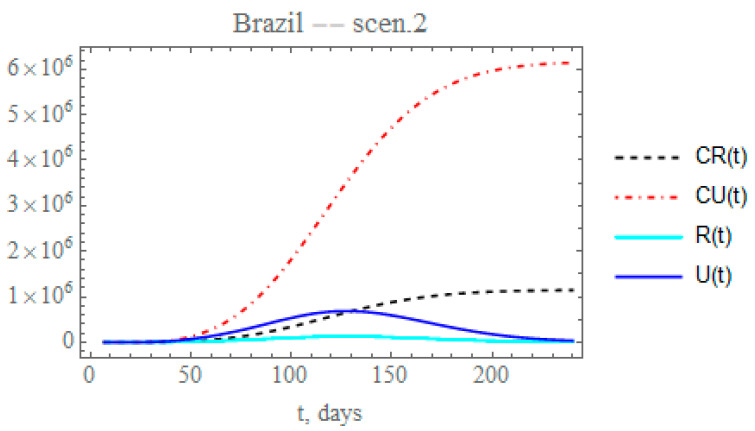
Scenario (ii) predictions for CR(t) (dashed black curve), CU(t) (dashed red curve), R(t) (solid cyan curve) and U(t) (solid blue curve) with the five estimated parameter values from the available dataset for Brazil from 25 February up to 29 March.

Therefore, the second scenario explores the implementation of a reduced decay of the transmission rate by assuming, after day *N*_2_ = 50 (Equation (10c)), that the value of μ here identified could be reduced by a factor of 0.2, thus around $\mu_{2}$ = 0.007793. The time variable transmission rate is then computed from Equation (12c) after t > *N*_2_. The changes in the accumulated reported and unreported cases, as shown in Figure 7, are quite significant. The predicted number of unreported symptomatic infectious cases is now much higher reaching after 240 days around 6.14454 × 10^6^ individuals, while the reported cases would reach 1.1436 × 10^6^ individuals, with a total of 7.28814 × 10^6^ symptomatic infected individuals and an impressive difference between reported and unreported cases, due to the maintained low value of $f_{max}$.

**Figure 8:**
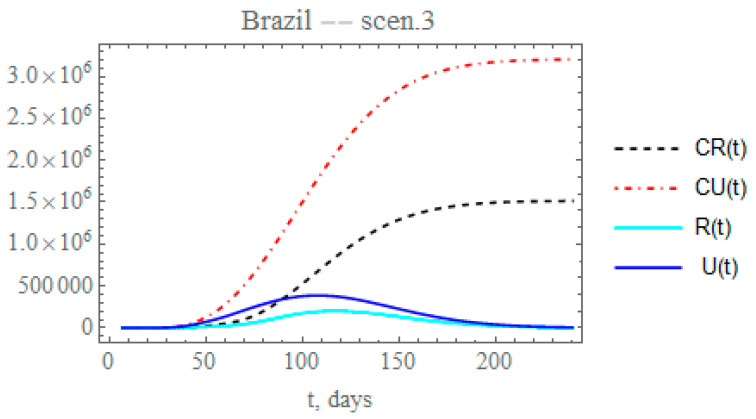
Scenario (iii) predictions for CR(t) (dashed black curve), CU(t) (dashed red curve), R(t) (solid cyan curve) and U(t) (solid blue curve) with the five estimated parameter values from the available dataset for Brazil from 25 February up to 29 March.

Though the reduced decay of the transmission rate shows a more realistic picture of the epidemy evolution after day 60, it is known from the Brazilian Ministry of Health site [31] that the PCR testing was markedly intensified after around day 60, corresponding to a total of about 3,820,000 applied tests by 26 June, and continuously increasing, as opposed to only 60,000 tests applied in the initial phase of the epidemy. Therefore, a more realistic picture of the ratio of reported to unreported cases is achieved by simultaneously increasing the partition factor. Through the third scenario, one can predict the consequences of reducing the exponential parameter that affects transmission rate, as in the previous scenario, and increasing by 25% the partition with respect to the initial factor ($f_{max2}=f_{0}\times 1.25$). This is simulated here by progressively increasing the partition factor, starting after day $N_{f2}$ = 64, taking an exponential parameter of $\mu_{f2}$ = 0.05, as expressed by Equation (12g), to allow for a gradual increase in testing. The changes in the accumulated and instantaneous reported and unreported symptomatic cases, as shown in Figure 8, are even more significant than in the second scenario (ii), Figure 7. The predicted number of accumulated unreported infectious cases is now reaching after 240 days around 3.2041 × 10^6^ individuals, while the reported cases would reach 1.51472 × 10^6^ individuals, with a marked decrease to a total of around 4.71882 × 10^6^ infectious symptomatic cases with respect to scenario (ii). Besides acting on the transmission rate along time, public health measures may also be effective in reducing the overall epidemic dynamics through the ratio of reported to the unreported infectious case, since the reported cases are, in general, directly isolated, and thus, interrupting the contamination path, as demonstrated in this third scenario, where a marked reduction in the total number of symptomatic infectious individuals from around 7.3 to 4.7 million is observed.

**Figure 9:**
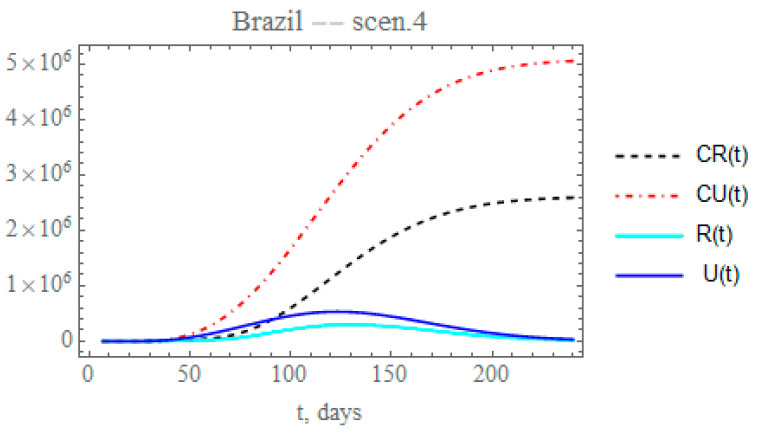
Scenario (iv) predictions for CR(t) (dashed black curve), CU(t) (dashed red curve), R(t) (solid cyan curve) and U(t) (solid blue curve) with the five estimated parameter values from the available dataset for Brazil from 25 February up to 29 March.

By analyzing the data on the percentual average circulation increase from day 50 to day 120 [29,30], assuming that the transmission rate can be approximately parametrized with the circulation, a reduction on the exponential factor of 0.177 could be expected, lower than that proposed in scenario (iii). Therefore, the fourth scenario examines the effect of further reducing the transmission factor coefficient, again starting at day N_2_ = 50, by a factor of 0.15, thus around *μ*_2_ = 0.005845. The fraction of reported and unreported infectious cases parameter, again reaches $f_{max2}=f_{0}\times 1.25=0.3796$, starting at $N_{f2}=64\;days\;\mathrm{with}\;\mu_{f2}=0.05,$ such as in the previous scenario. Therefore, Figure 9 shows the behavior of CR(t), R(t) and CU(t), U(t). The predicted number of unreported infectious cases would now reach, after 240 days, around 5.06029 × 10^6^ individuals, while the reported cases should reach 2.5922 × 10^6^ individuals, with an also marked reduction to a total of around 7.65249 × 10^6^ infectious cases. The predicted total number of infectious cases becomes even higher than that achieved in scenario (ii), illustrating the importance of an accurate estimation of the parameter that governs the temporal attenuation behavior of the transmission factor.

**Figure 10:**
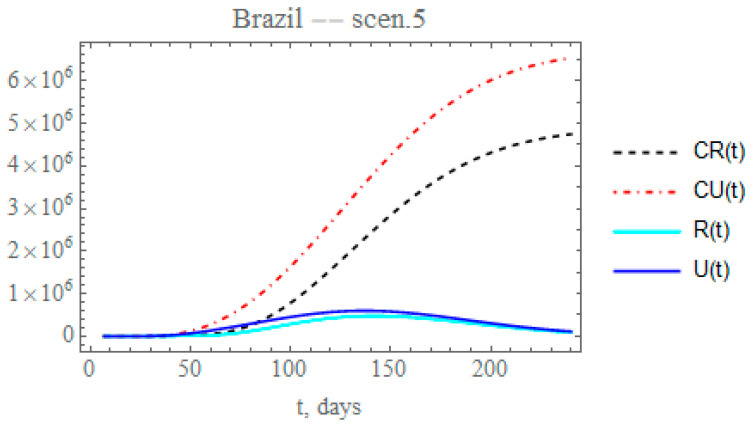
Scenario (v) predictions for CR(t) (dashed black curve), CU(t) (dashed red curve), R(t) (solid cyan curve) and U(t) (solid blue curve) with the five estimated parameter values from the available dataset for Brazil from 25 February up to 29 March.

By the time the final revision of this paper was being prepared, different cities and states throughout Brazil were taking partial isolation relaxation measures, while massive testing was being implemented in various locations. In light of the continental dimensions of the country and the fairly heterogeneous and non-simultaneous public health measures, the average overall response to these measures can be analyzed by simultaneously reducing the transmission exponential factor and increasing the maximum partition factor. Therefore, in the fifth scenario, the combination of public health measures affecting both the transmission rate and the conversion factor of unreported to reported cases is analyzed for Brazil. Let us consider that after day N_2_ = 50, the partial relaxation of social distancing leads to a reduction of 0.1 on μ value previously identified, thus around, $\mu_{2}$ = 0.0038964, and simultaneously increasing the fraction of reported and unreported infectious cases, to provide $f_{max2}=0.4555$, also starting after $N_{f2}=64\;days,$ with $\mu_{f2}$ = 0.05. The changes in the accumulated and instantaneous reported and unreported cases are shown in Figure 10. The predicted number of unreported infectious cases is now reaching after 240 days around 6.5372 × 10^6^ individuals, while the reported cases should reach 4.74799 × 10^6^ individuals, with a total of 11.2852 × 10^6^ infectious symptomatic cases, around 47.5% increase with respect to the previous scenario with lower transmission rates. Again, through the increased testing, a number of mild symptomatic cases were moved from the unreported to the reported cases evolution, thus moved to monitored isolation earlier, with some impact on the contagious chain, as can be observed through the final ratio of reported to unreported cases. In overall terms, the results for scenario (v) are markedly different from those for the previous scenario (iv), thus pointing out the importance of careful control of the social distancing relaxation measures, together with more intensive testing, to avoid such a marked change.

**Figure 11:**
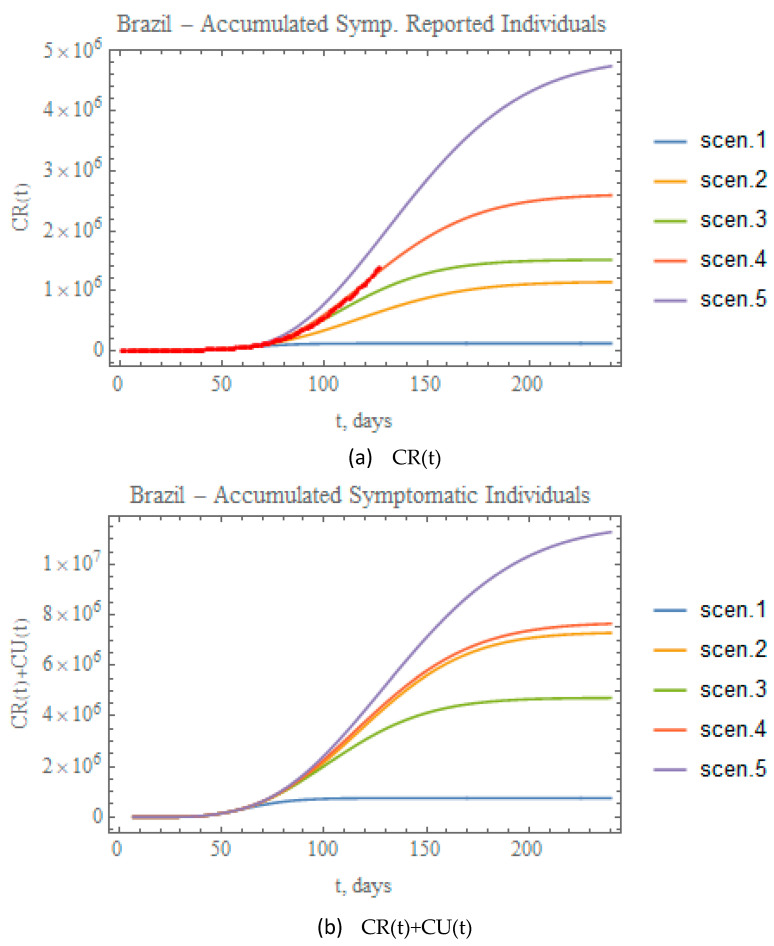
Comparative predictions for (**a**) CR(t), and (**b**) CR(t)+CU(t), for the five scenarios (i) to (v). (red dots in (**a**) show available data of CR(t) up to 29 June).

Figure 11a,b combine the data on accumulated reported and total (reported+unreported) infectious symptomatic individuals, respectively, for the predictions provided through the five scenarios here considered. Clearly, scenario (ii), though involving a more mild reduction in transmission rate attenuation coefficient, leads to significant numbers of total symptomatic infectious individuals, close to the values achieved for scenario (iv), essentially due to the fairly low partition factor that was retained in this case. On the other hand, when examining the fairly different total infectious cases curves for scenarios 4 and 5, it is clear that a proper combination of public health interventions, which would involve a more careful control on the relaxation of social distancing and further intensification of testing, should be implemented to achieve final results for scenario (v) closer to (iv), but with less strict circulation restrictions. The red dots in Figure 11a represent the actual accumulated reported cases data available up to 29 June. Clearly, these results are already closely represented by the conditions proposed for scenario (iv), without any previous parametric estimation. Such observation offers possible initial guesses for the model redefinition that shall be undertaken in the next section, based on the full dataset available at the completion of this work.

### 4.4. Model Redefinition: Brazil

The first model construction for Brazil in Section 4.2, employing only the first 35 days of data (up to 29 March) for the parametric estimation task, provided a fairly accurate prediction up to day 65 roughly to within 5% relative error on the accumulated reported cases evolution at this initial stage, which corresponds to the end of April. However, expected alterations on public health measures and behaviors that were initiated in the second half of April, have markedly modified the phenomenological pattern and the corresponding mathematical representation of the epidemy evolution. The scenarios analyzed in the previous section already anticipate the need for a model redefinition through a complementary inverse problem analysis that accounts for the added information from day 36 up to the last data available up to this work completion (day 127). The new parameters to be estimated correspond to those introduced through Equation (10c,d,f,g), not accounted for in the first model proposition and that were varied in the previous scenarios analysis, namely, $\mu_{2}$, $N_{2}$, $f_{max2}$, $\mu_{f2}$, and $N_{f2}$. As previously discussed, there is some prior information for the two date parameters, $N_{2}$ $and\;N_{f2}$, based on the fixed values adopted in the scenarios analysis (days 50 and 64, respectively), and therefore, an informative normal priori was employed for these two parameters. For the other three parameters, non-informative uniform priors were employed, with initial guesses provided by scenario (iv) previously discussed. Table 9 below summarizes the estimated values for the five parameters, together with the corresponding 99% confidence intervals.

**Figure 12:**
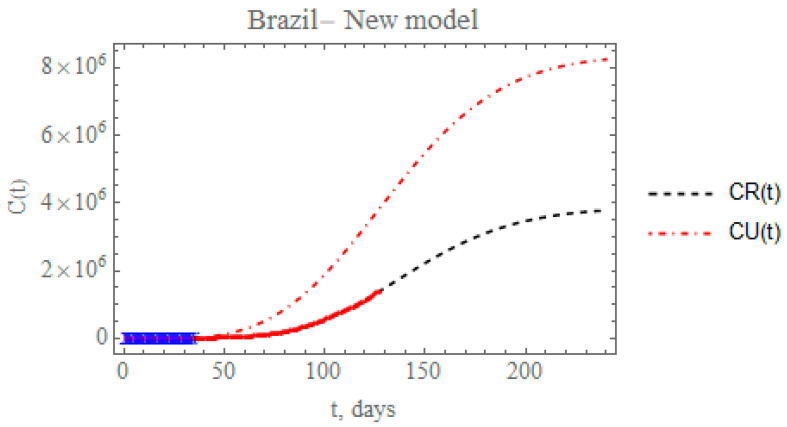
Prediction of the accumulated reported and unreported infectious, CR(t) and CU(t), with the five estimated parameter values from the available dataset for Brazil from 25 February up to 29 March (blue cross) together with the five new estimated parameter values from the data from 30 March to 29 June (red dots).

Figure 12 presents the predicted evolution of the accumulated reported and unreported infectious cases in Brazil, CR(t) and CU(t), from 25 February up to day 240, plotted as black and red dashed lines, respectively. Also shown in this figure are the blue crosses in the first portion of the available data, up to 29 March, which were employed in the estimation of the parameters in Table 7 that compose the initially proposed model. In addition, the red dots represent the second portion of the available data from 30 March until 29 June, that were now employed in the parametric estimation for the additional five parameters in Table 9. The predicted number of unreported infectious cases is now reaching after 240 days around 8.24397 × 10^6^ individuals, while the reported cases reach 3.78342 × 10^6^ individuals, with a total of 12.0274 × 10^6^ infectious symptomatic cases. From the identified value of the partition factor in Table 9, which is still well below the value encountered from the China analysis, there is space for further intensification of the testing campaigns throughout Brazil, towards a reduction on the total number of symptomatic infectious individuals in the long term.

**Figure 13:**
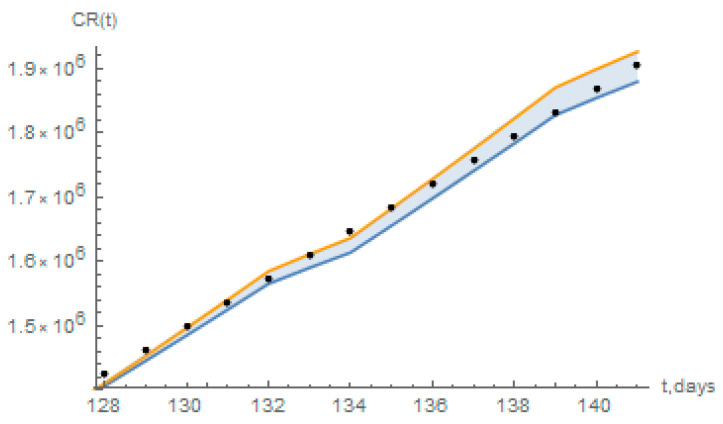
Short term prediction of the accumulated reported infectious, CR(t), with the redefined model for Brazil (black dots), and the estimated 95% confidence intervals for a logistic growth model as computed from Reference [32].

The short term predictions from the redefined model have also been compared to a statistical simulation that employs a basic logistic growth model [32], essentially based on the data for reported infectious individuals. From the application in Reference [32] it has been computed from the data up to 29 June, the values of accumulated reported cases estimated from 30 June (day 128) up to 13 July (day 141), with 95% confidence intervals, as shown in Figure 13 below, together with the predictions of the present redefined model. As can be seen, the present short-term predictions for the days 128 to 141 mostly fall within the 95% confidence interval estimates of the logistic growth model in Reference [32].

Finally, Table 10 summarizes the predicted accumulated reported, unreported, and total symptomatic infectious individuals at day t = 240 for each of the five scenarios previously considered and for the redefined model with the full data set for Brazil (Appendix A). Also shown is the maximum value of the number of instantaneous reported cases, R_max_, and the time of its occurrence, t_max_. Once more, it is quite evident that the first built model with the initial 35 data points could only capture the phenomenological characteristics of the epidemy up to a certain date (roughly up to day 65, as previously discussed). Then, the combination of circulation augmentation and intensified testing led to a markedly different evolution, later confirmed by the redefined model. The marked differences between these two models are observable through the predicted total number of symptomatic individuals at day 240, varying from around 0.75 million in the first model to 12.03 million in the second one. Moreover, the peaks of the instantaneous reported infectious individuals varied from around day 70 in the first model to about day 147 in the second one. In between these two models, one can observe from Table 10, the number of accumulated symptomatic individuals at 240 days and the reported infections peaks predictions for the other four scenarios. Again we point out the importance of the intensified testing and the proper isolation of those positively tested in the epidemy evolution control, which is evident in the total number of symptomatic individuals, being much lower for scenario (iii) with respect to (ii), while the peak of reported symptomatic cases is noticeably advanced in time (roughly from day 127 down to day 117).

## 5. Conclusions

The present work implements a mixed analytical-statistical inverse problem analysis in the prediction of epidemy evolution, with a focus on the COVID-19 progression in Brazil. A SIRU-type model is adopted, which inherently accounts for the unreported symptomatic infectious individuals. The following aspects can be highlighted in the present study:

- Time-variable functional parametrizations are proposed for the transmissivity and for the partition of reported and unreported symptomatic cases, which allow for the consideration of public health measures along the epidemy evolution, such as reducing or increasing circulation and intensifying PCR testing while isolating the positively tested individuals.
- The inverse problem analysis is based on the combination of an analytical parametric estimation for the early phase epidemic exponential behavior with a Bayesian inference approach for a longer following period that encompasses the initial public health interventions to control the epidemics.
- The evolution of the COVID-19 epidemy in China is considered for validation purposes, by taking the first part of the dataset of accumulated reported infectious individuals to estimate the related parameters, and retaining the rest of the evolution data for direct comparison with the predicted results, with excellent agreement.
- The same approach is applied to the Brazilian case, this time employing an initial portion of the available time series for the parametric estimates up to day 35, and then offering a validation of the evolution prediction through the initially available remaining dataset up to day 60 (23 April).
- This first constructed model provides fairly accurate predictions up to day 65 below 5% relative deviation, when the data starts detaching from the theoretical curve.
- Afterwards, some public health intervention measures are critically examined through five different scenarios, permitting the inspection of their impact on the overall dynamics of the disease proliferation. It was observed that a combination of careful control of the social distancing relaxation and sanitary habits, together with more intensive testing for isolation of symptomatic cases, would be necessary to achieve the overall control of the disease and avoid a second more strict social distancing intervention.
- The model is then redefined employing the full data set at the completion of this work (day 36 to 127), and new parameters were identified to describe this second phase of the epidemy. Predictions of the reported and unreported symptomatic cases are then offered with the redefined model and briefly compared with a statistical simulation that employs a basic logistic growth model for the short term epidemy behavior.

Further improvement on the modeling is envisioned by enriching the model with latency effects, age structure discrimination, spatial demographic distribution dependence, and recovery factor differentiation among isolated and non-isolated patients.

## Figures and Tables

**Table 1:** Summary of the estimated parameters on each inverse problem analysis.

Case	Parameter under Estimation	Data Range Used in Inverse Problem
China		
case 1: CH3p	$f_{0},\;\mu ,\tau_{0}$	19 January up to 17 February
case 2: CH5p-full	$f_{0},\;\mu ,\tau_{0},1/\nu ,\;1/\eta$	19 January up to 16 April
Brazil		
case 3: BR5p	$f_{0},\;\mu ,\;\tau_{0},\;f_{max},\;N_{f}$	25 February up to 29 March
case 4: BR5p	$\mu_{2}$, $N_{2}$, $f_{max2}$, $\mu_{f2}$, $N_{f2}$	30 March up to 26 June

**Table 2:** Prior distributions and initial guesses for the parameters to be estimated $f_{0},\;\mu ,\;\mathrm{and}\;\tau_{0}$ (Wuhan, China).

Case 1: CH3p			
Parameter	Prior Distribution	Initial Guess	Source
$f_{0}$	U[0,1]	0.5	estimated
μ	U[0,5]	0.1	estimated
$\tau_{0}$	$U\left[0,\;1\times 10^{-6}\right]$	$4.478\times 10^{-8}$	estimated
*S_0_*	11.0 × 10^6^		fixed
$t_{0}$	5.04617		fixed
1/ν	7 days		fixed
1/η	7 days		fixed
N	25		fixed
$\chi_{1}$	0.14936		fixed
$\chi_{2}$	0.37680		fixed
$\chi_{3}$	1.0		fixed

**Table 3:** Estimated values and 99% confidence intervals for three parameters,$f_{0},\;\mu ,\mathrm{and}\;\tau_{0}$ (Wuhan, China).

Case 1: CH3p		
Parameter	Estimated Values	99% Confidence Interval
$f_{0}$	0.780719	[0.77956, 0.7818]
μ	0.135635	[0.135219, 1.136153]
$\tau_{0}$	$4.47793\times 10^{-8}$	$\left[4.47793\times 10^{-8},\;4.47793\times 10^{-8}\right]$

**Table 4:** Prior distributions and initial guesses for the five parameters to be estimated, $f_{0},\;\mu ,\;\tau_{0},\;1/\nu \;\mathrm{and}\;1/\eta$, (Wuhan, China)

Case 2: CH5p			
Param.	Prior Distribution	Initial Guess	
$f_{0}$	U[0,1]	0.8	estimated
μ	U[0,5]	0.131	estimated
$\tau_{0},\;$	$U\left[0,\;1\times 10^{-6}\right]$	$4.4779\times 10^{-8}$	estimated
1/ν	N[5.2, σ=2.1] (Min = 1, Max = 21)	7	estimated
1/η	N[10.4,σ=2.6] (Min = 1, Max = 21)	7	estimated
*S_0_*	11.0 × 10^6^		fixed
$t_{0}$	5.04617		fixed
N	25		fixed
$\chi_{1}$	0.14936		fixed
$\;\chi_{2}$	0.37680		fixed
$\;\chi_{3}$	1.0		fixed

**Table 5:** Estimated values and 99% confidence intervals for five parameters, $f_{0},\;\mu ,\;\tau_{0},\;1/\nu \;\mathrm{and}\;1/\eta$ (Wuhan, China).

Case 2: CH5p		
Parameter	Estimated Values	99% Confidence Interval
$f_{0}$	0.718491	[0.711595, 0.723138]
μ	0.132032	[0.131789, 0.13227]
τ0	$4.47793\times 10^{-8}$	$\left[4.47793\times 10^{-8},\;4.47793\times 10^{-8}\right]$
1/ν	6.20798	[6.12574, 6.25764]
1/η	11.2784	[10.4379, 12.3593]

**Table 6:** Prior distributions and initial guesses for the five parameters to be estimated, $f_{0},\;\mu ,\;\tau_{0},\;f_{max},\;N_{f}\;$ (Brazil).

Case 3—BR5p			
Param.	Prior Distribution	Initial Guess	Source
*f*_0_	U[0,1]	0.300	estimated
μ	U[0, 5]	0.04	estimated
$\tau_{0}$	$U\left[0,\;1\times 10^{-6}\right]$	$1.66755\times 10^{-9}$	estimated
*f_max_*	*U*[0, 1]	0.165	estimated
*N_f_*	*U*[10, 35]	30.5	estimated
1/ν	6.20798 days		fixed
1/η	11.2784 days		fixed
S_0_	211.3 × 10^6^		fixed
$t_{0}$	6.90514		fixed
N	21		fixed
*μ_f_*	10		fixed
*χ_1_*	0.42552		fixed
$\chi_{2}$	0.293696		fixed
*χ_3_*	3.2335		fixed

**Table 7:** Estimated values and 99% confidence intervals for five parameters $f_{0},\;\mu ,\;\tau_{0},\;f_{max},\;\mathrm{and}\;N_{f}\;$ (Brazil).

Case 3—BR5p		
Parameter	Estimated Values	99% Confidence Interval
$f_{0}$	0.303671	[0.302624, 0.304697]
μ	0.0389639	[0.0388438, 0.0390961]
$\tau_{0}$	$1.66755\times 10^{-9}$	$\left[1.66755\times 10^{-9},\;1.66755\times 10^{-9}\right]$
$f_{max}$	0.156734	[0.156146, 0.157217]
$N_{f}$	30.4197	[30.3522, 30.4915]

**Table 8:** Input data in each scenario for COVID-19 epidemy evolution in Brazil.

Fixed Parameters					
Scenario	All-(i) to (v)				
$f_{0}$	0.303671				
*μ*	0.0389639				
*N*	21				
$\tau_{0}$	$1.66755\times 10^{-9}$				
$f_{max}$	0.156734				
$\mu_{f}$	2				
$N_{f}$	30.4197				
1/ν	6.20798 days				
1/η	11.2784 days				
*S_0_*	$211.3\times 10^{6}$				
*t*_0_	6.90514				
$\chi_{1}$	0.42552				
$\chi_{2}$	0.293696				
$\chi_{3}$	3.2335				
**Changing Parameters: (50–240th day)**					
**Scenario**	**(i)**	**(ii)**	**(iii)**	**(iv)**	**(v)**
$\mu_{2}$	0	μ×0.2	μ×0.2	μ×0.15	μ×0.1
$N_{2}$	-	50	50	50	50
$f_{max2}$	-	-	$f_{0}\times 1.25$	$f_{0}\times 1.25$	$f_{0}\times 1.5$
$\mu_{f2}$	0	0	0.05	0.05	0.05
$N_{f2}$	-	64	64	64	64

**Table 9:** Estimated values and 99% confidence intervals for five parameters, $\mu_{2}$, $N_{2}$, $f_{max2}$, $\mu_{f2}$, and $N_{f2}$ (Brazil).

Case 4—BR5p		
Parameter	Estimated Values	99% Confidence Interval
$\mu_{2}$	0.00489914	[0.00489891, 0.00489951]
$N_{2}$	50.2273	[50.2132, 50.2378]
$f_{max2}$	0.384824	[0.382447,0.386403]
$\mu_{f2}$	0.0200351	[0.020032, 0.0200378]
$N_{f2}$	62.1399	[61.971, 62.3343]

**Table 10:** Summary of predicted accumulated reported and unreported cases at day 240 for the scenarios and maximum value and time of instantaneous reported infectious cases (Brazil).

Scenario	CR(240) × 10^6^	CU(240) × 10^6^	[CR(240)+CU(240)] × 10^6^	R_max_	t_max_
(i)	0.11919	0.633698	0.752888	26755	70.2
(ii)	1.14360	6.14454	7.28814	127209	127.4
(iii)	1.51472	3.20410	4.71882	201318	116.9
(iv)	2.59220	5.06029	7.65249	300014	129.5
(v)	4.74799	6.53720	11.2852	479487	141.9
New model	3.78342	8.24397	12.0274	403964	146.9

## Nomenclature

CR(t)	cumulative number of reported infectious symptomatic cases at time *t*
CU(t)	cumulative number of unreported infectious symptomatic cases at time *t*
DR(t)	daily number of reported infectious symptomatic cases at time *t*
f(t)	time-variable fraction of asymptomatic infectious cases who become reported symptomatic infectious cases
$f_{0}$	initial fraction of asymptomatic infectious cases who become reported symptomatic infectious cases
$f_{max}$	second value of fraction of asymptomatic infectious cases who become reported symptomatic infectious cases
$f_{max2}$	third value of fraction of asymptomatic infectious cases who become reported symptomatic infectious cases
I(t)	number of asymptomatic infectious individuals at time *t*
I_0_	asymptomatic infectious individuals at initial time $t_{0}$
N	time in days of application of the first public health intervention that changes the transmission rate
$N_{2}$	time in days of application of the second public health intervention that changes the transmission rate
$N_{f}$	time in days for application of the first public health intervention that changes the fraction of asymptomatic infectious cases that become reported symptomatic infectious cases
$N_{f2}$	time in days for application of the second public health intervention that changes the fraction of asymptomatic infectious cases that become reported symptomatic infectious cases
NP	total number of parameters in the estimation algorithm
M	total number of measured data used in the estimation
*R*(*t*)	number of reported symptomatic infectious individuals at time *t*
R_0_	reported symptomatic infectious individuals at initial time $t_{0}$
P	vector of parameters under estimation (P_1_, P_2_, …, P_NP_)
S(t)	number of individuals susceptible to infection at time *t*
S_0_	number of individuals susceptible to infection at initial time $t_{0}$
t	time variable, in days
$t_{0}$	beginning date of the epidemic, in days
U(t)	number of unreported symptomatic infectious individuals at time *t*
U_0_	unreported symptomatic infectious individuals at initial time $t_{0}$
Y	vector of measured data (Y_m_ at time t_m_, m = 1, …, M)
Greek Symbols	
1/η	average time in days that the symptomatic infectious cases remain with symptoms
1/ν	average time in days that the asymptomatic infectious cases remain asymptomatic
ν1	rate at which asymptomatic infectious cases become reported symptomatic infectious cases
ν2	rate at which asymptomatic infectious cases become unreported symptomatic infectious cases
μ	argument of the τ(t) function
$\mu_{2}$	argument of the τ(t) function
$\mu_{f}$	argument of the *f*(*t*) function
$\mu_{f2}$	argument of the *f*(*t*) function
π(Y)	marginal probability density
π(Y|P)	likelihood function
$\pi_{posterior}$	posterior probability density
$\pi_{prior}$	prior probability density
τ(t)	time-variable transmission rate
$\tau_{0}$	initial amplitude of the *τ*(*t*) function
$\chi_{1}$	fitting parameter in the early exponential phase of the epidemy, Equation (7a)
$\chi_{2}$	fitting parameter in the early exponential phase of the epidemy, Equation (7a)
$\chi_{3}$	fitting parameter in the early exponential phase of the epidemy, Equation (7a)

## Appendix A

**Table A1:** Data for Brazil—accumulated reported cases, CR(t), and casualties.

DATE	Day	Deaths	Infected	DATE	Day	Deaths	Infected
24 February 2020	1	0	0	28 April 2020	65	5050	72,149
25 February 2020	2	0	1	29 April 2020	66	5484	78,416
26 February 2020	3	0	1	30 April 2020	67	5912	85,507
27 February 2020	4	0	1	1 May 2020	68	6354	91,604
28 February 2020	5	0	1	2 May 2020	69	6750	96,559
29 February 2020	6	0	2	3 May 2020	70	7044	101,227
1 March 2020	7	0	2	4 May 2020	71	7321	107,780
2 March 2020	8	0	2	5 May 2020	72	7921	114,715
3 March 2020	9	0	2	6 May 2020	73	8536	125,218
4 March 2020	10	0	3	7 May 2020	74	9146	135,106
5 March 2020	11	0	8	8 May 2020	75	9897	145,328
6 March 2020	12	0	13	9 May 2020	76	10,627	155,939
7 March 2020	13	0	19	10 May 2020	77	11,123	162,699
8 March 2020	14	0	25	11 May 2020	78	11,519	168,331
9 March 2020	15	0	25	12 May 2020	79	12,400	177,589
10 March 2020	16	0	34	13 May 2020	80	13,149	188,974
11 March 2020	17	0	52	14 May 2020	81	13,993	202,918
12 March 2020	18	0	77	15 May 2020	82	14,817	218,223
13 March 2020	19	0	151	16 May 2020	83	15,633	233,142
14 March 2020	20	0	151	17 May 2020	84	16,118	241,080
15 March 2020	21	0	200	18 May 2020	85	16,792	254,220
16 March 2020	22	0	234	19 May 2020	86	17,971	271,628
17 March 2020	23	1	346	20 May 2020	87	18,859	291,579
18 March 2020	24	4	529	21 May 2020	88	20,047	310,087
19 March 2020	25	7	640	22 May 2020	89	21,048	330,890
20 March 2020	26	11	970	23 May 2020	90	22,013	347,398
21 March 2020	27	18	1178	24 May 2020	91	22,666	363,211
22 March 2020	28	25	1546	25 May 2020	92	23,473	374,898
23 March 2020	29	34	1924	26 May 2020	93	24,512	391,222
24 March 2020	30	46	2247	27 May 2020	94	25,598	411,821
25 March 2020	31	57	2433	28 May 2020	95	26,754	438,238
26 March 2020	32	77	2985	29 May 2020	96	27,878	465,166
27 March 2020	33	92	3417	30 May 2020	97	28,834	498,440
28 March 2020	34	111	3904	31 May 2020	98	29,314	514,200
29 March 2020	35	136	4256	1 June 2020	99	29,937	526,447
30 March 2020	36	159	4589	2 June 2020	100	31,199	555,383
31 March 2020	37	201	5744	3 June 2020	101	32,548	584,016
1 April 2020	38	242	6840	4 June 2020	102	34,021	614,941
2 April 2020	39	299	7911	5 June 2020	103	35,026	645,771
3 April 2020	40	359	9095	6 June 2020	104	35,930	672,846
4 April 2020	41	432	10,298	7 June 2020	105	36,455	691,758
5 April 2020	42	486	11,132	8 June 2020	106	37,134	707,412
6 April 2020	43	555	12,066	9 June 2020	107	38,406	739,503
7 April 2020	44	679	13,862	10 June 2020	108	39,680	772,416
8 April 2020	45	814	16,037	11 June 2020	109	40,919	802,828
9 April 2020	46	943	17,886	12 June 2020	110	41,828	828,810
10 April 2020	47	1065	19,730	13 June 2020	111	42,720	850,514
11 April 2020	48	1131	20,818	14 June 2020	112	43,332	867,624
12 April 2020	49	1225	22,206	15 June 2020	113	43,959	888,271
13 April 2020	50	1333	23,604	16 June 2020	114	45,241	923,189
14 April 2020	51	1541	25,371	17 June 2020	115	46,510	955,377
15 April 2020	52	1745	28,532	18 June 2020	116	47,748	978,142
16 April 2020	53	1933	30,449	19 June 2020	117	48,954	1032,913
17 April 2020	54	2143	33,759	20 June 2020	118	49,976	1067,579
18 April 2020	55	2359	36,739	21 June 2020	119	50,617	1085,038
19 April 2020	56	2468	38,688	22 June 2020	120	51,271	1,106,470
20 April 2020	57	2584	40,616	23 June 2020	121	52,645	1,145,906
21 April 2020	58	2751	43,131	24 June 2020	122	53,830	1,188,631
22 April 2020	59	2917	45,976	25 June 2020	123	54,971	1,228,114
23 April 2020	60	3320	49,711	26 June 2020	124	55,961	1,274,974
24 April 2020	61	3688	53,448	27 June 2020	125	57,070	1,313,667
25 April 2020	62	4047	58,973	28 June 2020	126	57,622	1,344,143
26 April 2020	63	4244	62,208	29 June 2020	127	58,314	1,368,195
27 April 2020	64	4554	66,541				
